# Supplementary material for: Atypical chemoreceptor arrays accommodate high membrane curvature
Source: Nat Commun. 2020 Nov 13;11:5763. doi: 10.1038/s41467-020-19628-6 (PMC7666581; doi:10.1038/s41467-020-19628-6)
Supplement: Supplementary file 2 — Supplementary Information [file 41467_2020_19628_MOESM2_ESM.pdf]

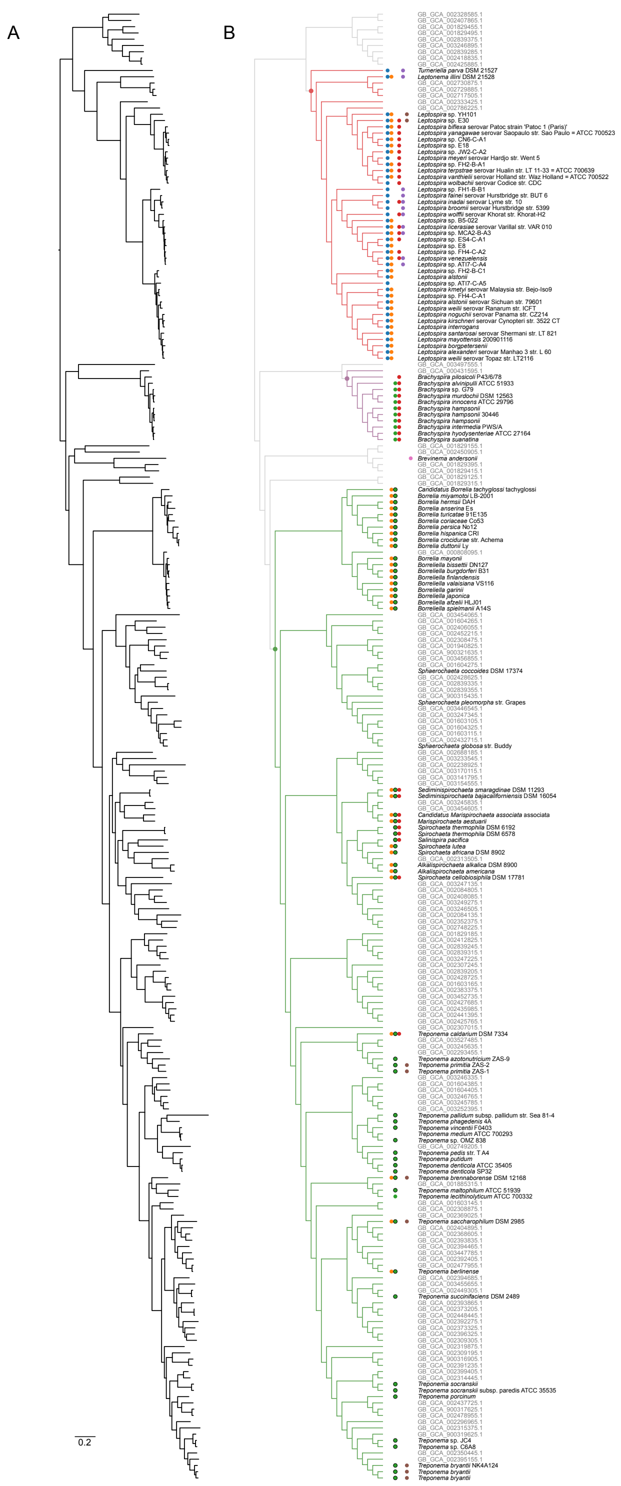

Fig. S1 Complete chemosensory class profile of Spirochaetota. The three major taxonomy classes are colored: Leptospirae (red), Brachyspirae (purple) and Spirochaetia (green). Species found in MiST3 are represented by their species and strain name. The profile shows the presence of the chemotaxis classes F1(blue), F8(orange), F2 (green), F7(red), F5 (purple), ACF (brown) and TFP (pink). The systems with CheW-$CheR_{like}$ are marked with a black outline. Scale bar represents the average number of substitutions per site. An additional high-resolution figure is separately available.

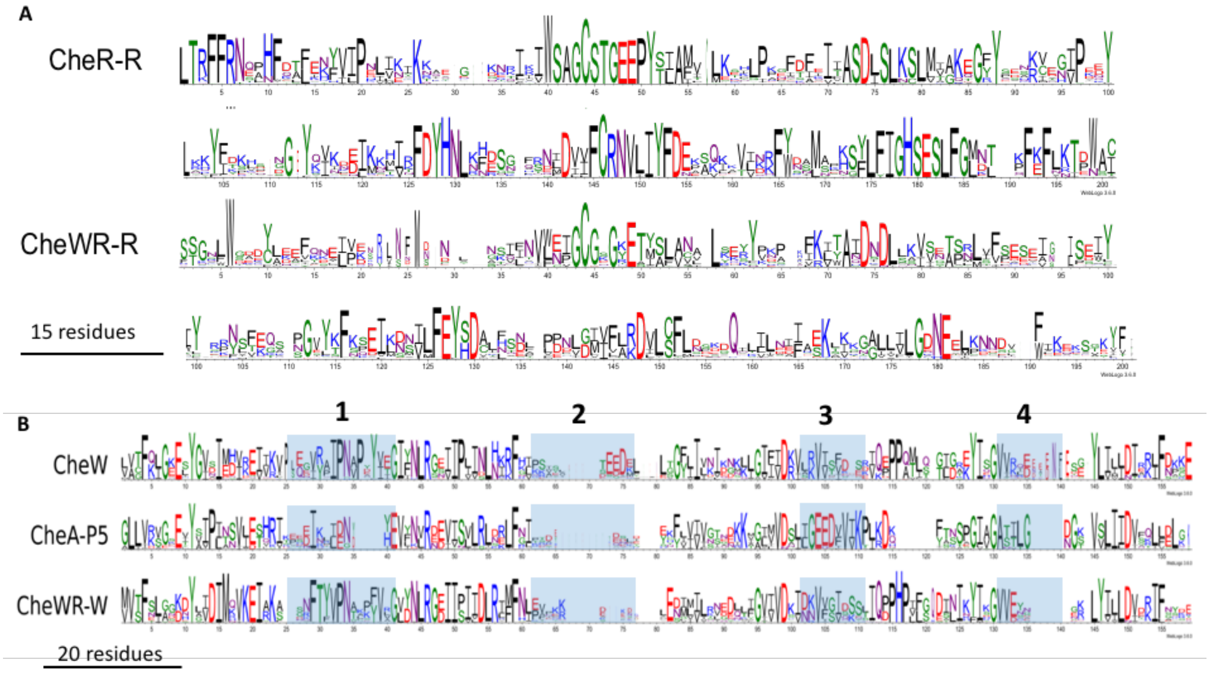

Fig. S2 (A) Sequence logo of representative sequences of the two groups of CheR-containing proteins in the F2 system. (B) Sequence logo of representative sequences of the three groups of CheW-containing proteins in the F2 system. Blue boxes denote the location of variable regions identified in the *Td* CheW and P5 homologs. In these locations, all regions possess unique conserved residues with the exception of Region 2, which is not at the CheA:CheW ring interfaces. An additional high-resolution figure is separately available.

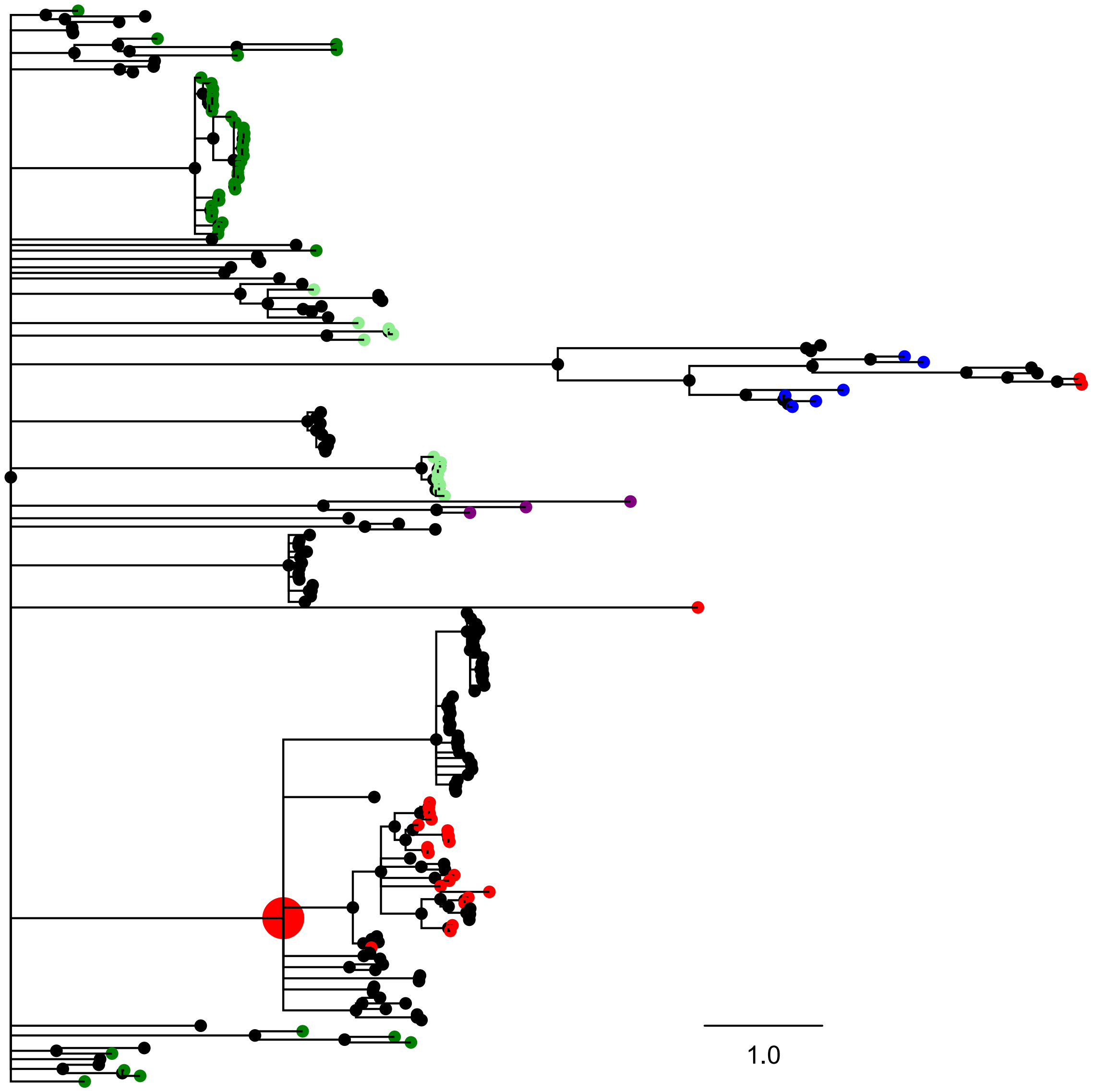

Fig. S3 Phylogenetic tree of a non-redundant set of CheW protein sequences in genomes with at least one CheA-F2. The initial classification of CheW classes are mapped to the tree nodes: F2 (red), F5 (purple), F7 (light green), F8 (green) and ACF (blue). The clustering of the CheW classified as class F2 suggests a last common ancestor of CheW-F2 sequences (larger red internal node). This clustering allows us to extrapolate the conservative selection of F2 sequences to other CheW proteins in the cluster to get a more inclusive set. Scale bar represents the average number of substitutions per site.

A

Td strain	No. Cells/Arrays	No. Particles	Average resolution
WT	11	728	16 Å
Δ2498	10	894	16 Å
Δ2498Δ2496	10	554	25 Å
ΔCheR$_{like}$	5	194	46 Å

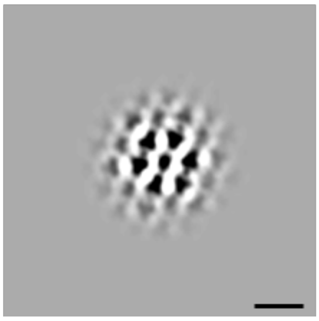

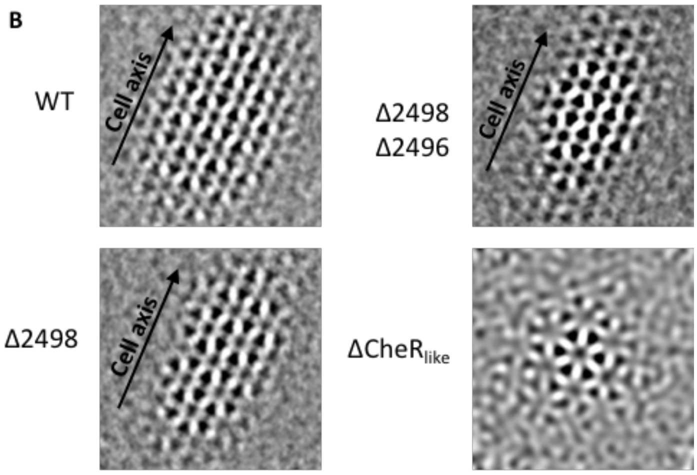

Fig. S4 (A) Top: The number of arrays and particles selected to generate sub-tomogram averages for each *Td* strain, and the average resolution of the sub-tomogram averages. Resolution values were calculated at FSC = 0.3 in a masked region that contains the single central receptor hexagon and CheA:CheW ring. Bottom: Example of the masked region used for resolution calculation (WT map). Scale bar is 12 nm. (B) Sub-tomogram averages reveal the orientation of the chemotaxis arrays with respect to the cell axis. Intriguingly, the arrays have a preferred orientation in the cells and this orientation is conserved in WT and the Δ2498 and Δ2498Δ2496 deletion mutants. The small size of arrays in the ΔCheR$_{like}$ strain inhibits direct observation of the cell axis.

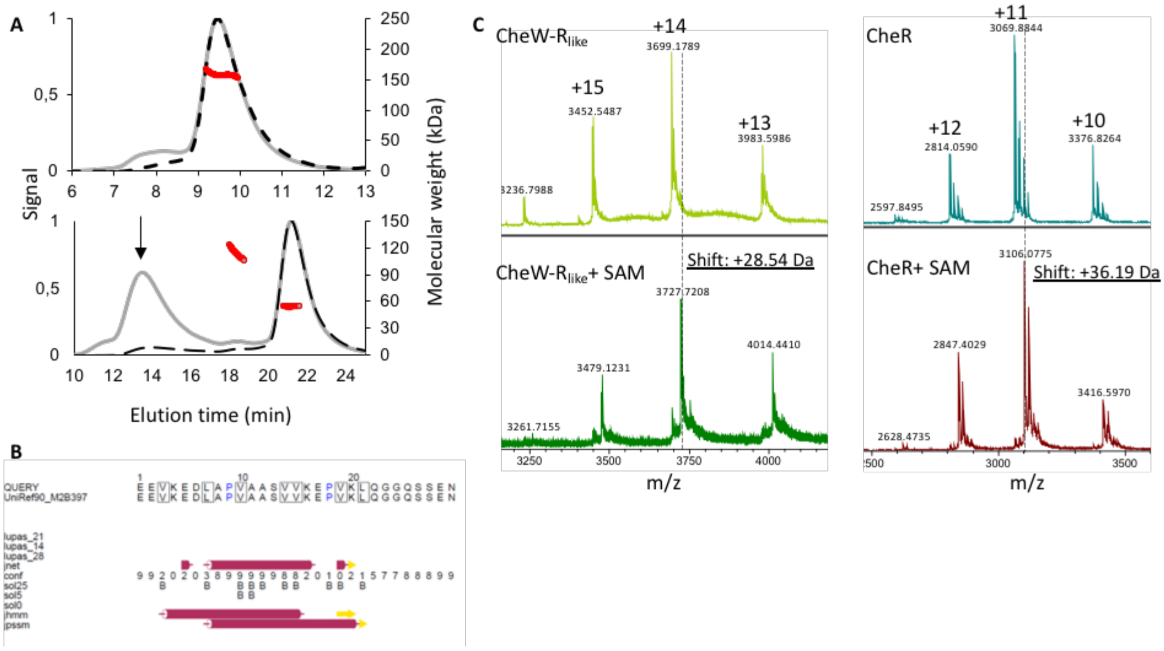

Fig. S5 (A) SEC-MALS of *Td* CheA and CheW-R$_{like}$. Top: CheA forms a dimer in solution. Bottom: The CheW-R$_{like}$ protein is primarily present as a monomer, but associates into a small amount of dimer. There is also a small amount of aggregates present (black arrow). The dRI trace is a black dashed line. The LS trace is grey. (B) The CheW-CheR$_{like}$ linker is predicted to form a single alpha helix flanked by unordered regions (Jpred). (C) Native mass spectrometry (ESI-MS) experiments indicate that CheW-R$_{like}$ (left) and the *Td* classical CheR protein (right) bind SAM (398.44 Da) in a 1:1 stoichiometric ratio. Example shifts in the spectra are denoted by black dashed lines and match expectations for 1:1 binding. The sequence derived mass of CheW-CheR$_{like}$ and CheR is 51.784 kDa and 33.802 kDa, respectively. Source data are provided as a Source Data file.

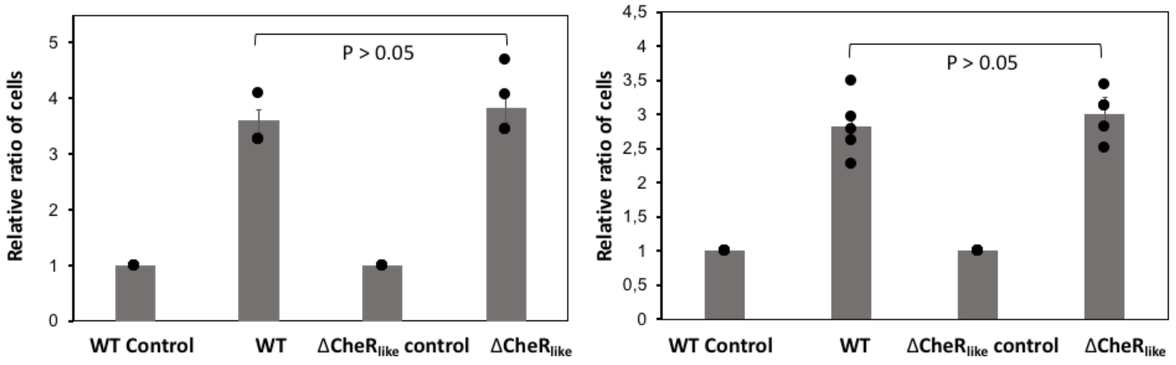

Fig. S6 Capillary chemotaxis assay of *T. denticola* wild-type and $\Delta CheR_{like}$ strain using two chemoattractants: hemin (left) and glucose (right). The $\Delta CheR_{like}$ strain is missing only the $CheR_{like}$ domain of CheW-$CheR_{like}$. For the non-gradient control, both the capillary tubes and bacterial suspensions contained chemoattractants. Differences in chemoattraction of WT and $\Delta CheR_{like}$ strains are statistically insignificant using a two-tailed null hypothesis significance test ($p > 0.05$). Results are expressed as the mean of cell numbers ± standard error of the mean (SEM) from five capillary tubes. The data are normalized so that the control samples are equal to 1. The normalized cell ratio values for individual samples (capillary tubes) are shown as black dots. Source data are provided as a Source Data file.

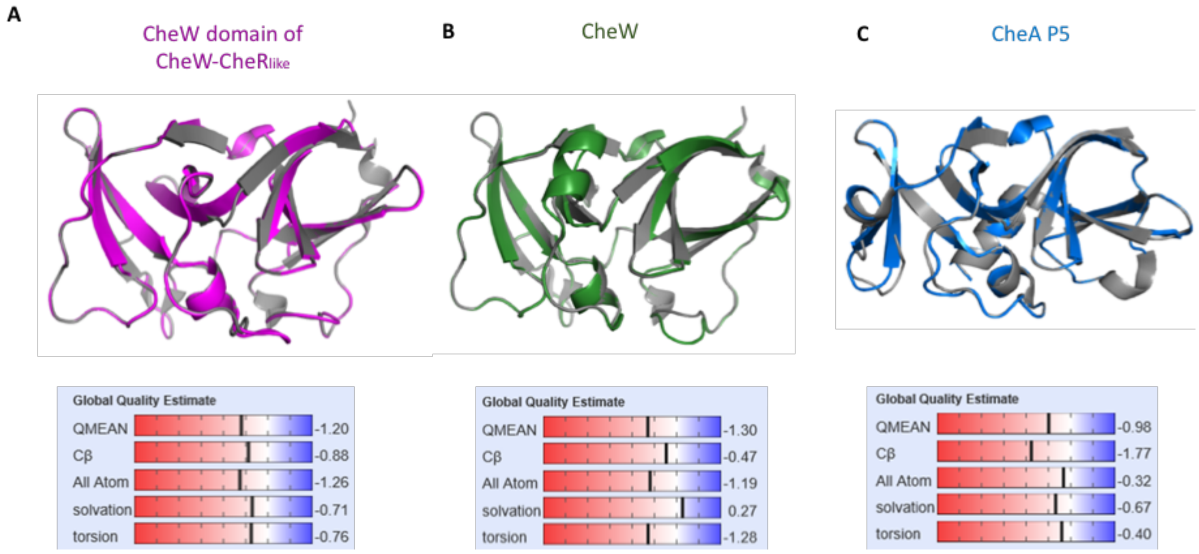

Fig. S7 Homology models of *Td* CheW domains and CheA P5. (A) A homology model of the CheW domain of CheW-CheRlike using *Thermoanaerobacter tengcongensis* CheW (PDB ID: 2QDL) as the template. (B) A homology model of the classical *Td* CheW using *Thermoanaerobacter tengcongensis* CheW (PDB ID: 2QDL) as the template. (C) A homology model of CheA P5 using *E. coli* CheA P5 (PDB ID: 6S1K) as the template.

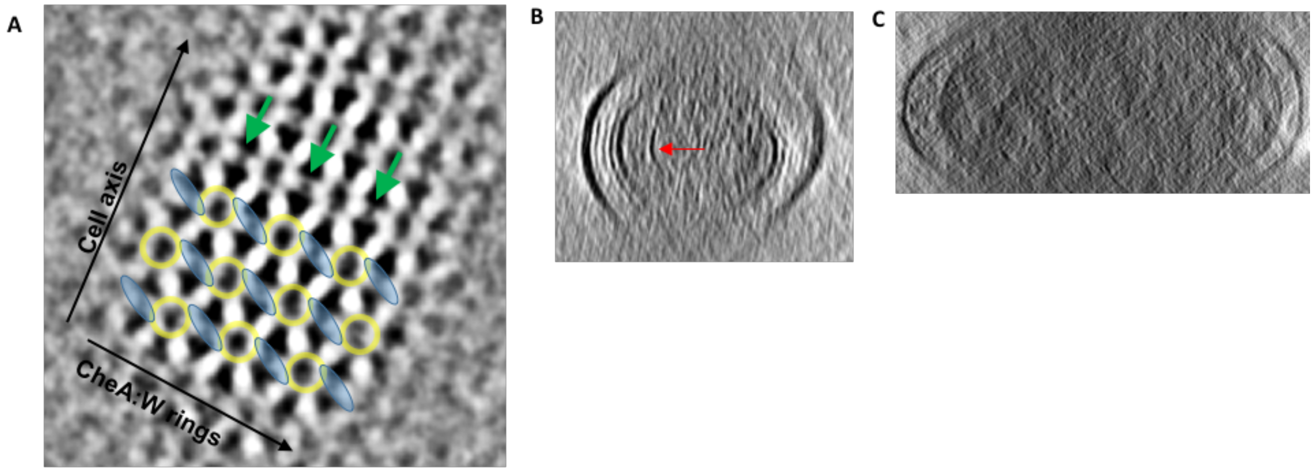

Fig. S8 (A) Sub-tomogram averaging of *Td* WT and mutant strains reveal that linked CheA:CheW rings (yellow) run perpendicular to the cell axis via a strict linear orientation of CheA (blue). Dimerization of CheA at the P3 domain (green arrows) links the rings together. Averages from WT *Td* are illustrated here but apply to all strains. (B) The curvature of the inner membrane of *Td* at chemotaxis arrays in the reconstructions is 35.8 +/- 6.6 /µm (270 Å radius). The curvature of the CheA:CheW baseplate (red arrow) is 65.6 +/- 19 /µm (152 Å radius). (C) *V. cholerae* minicells have an inner membrane curvature of 9.15 +/- 4.5 /µm (radius 1092 Å).

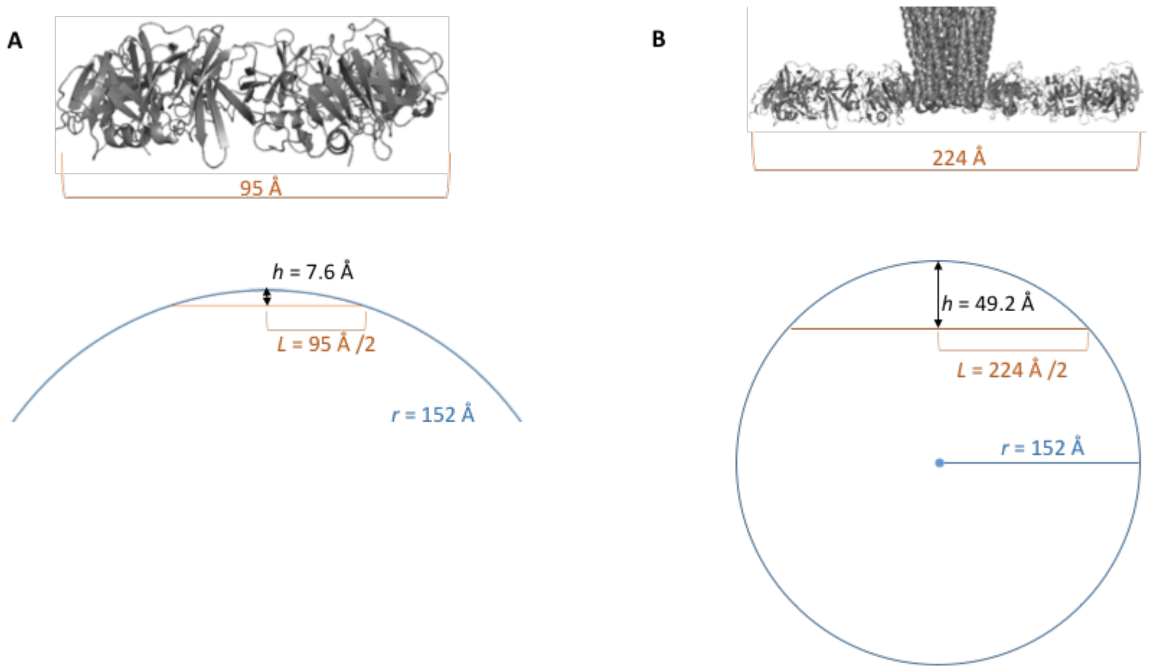

Fig. S9 Modeling of CheA:CheW rings to the curvature of the *Td* baseplate. (A) For single CheA:CheW rings to follow the baseplate curvature, it must bend by an average of 7.6 Å toward the membrane. (B) In order for two linked CheA:CheW rings to run perpendicular to the cell axis, the center of the rings (P3) must bend by 49.2 Å toward the cell membrane.

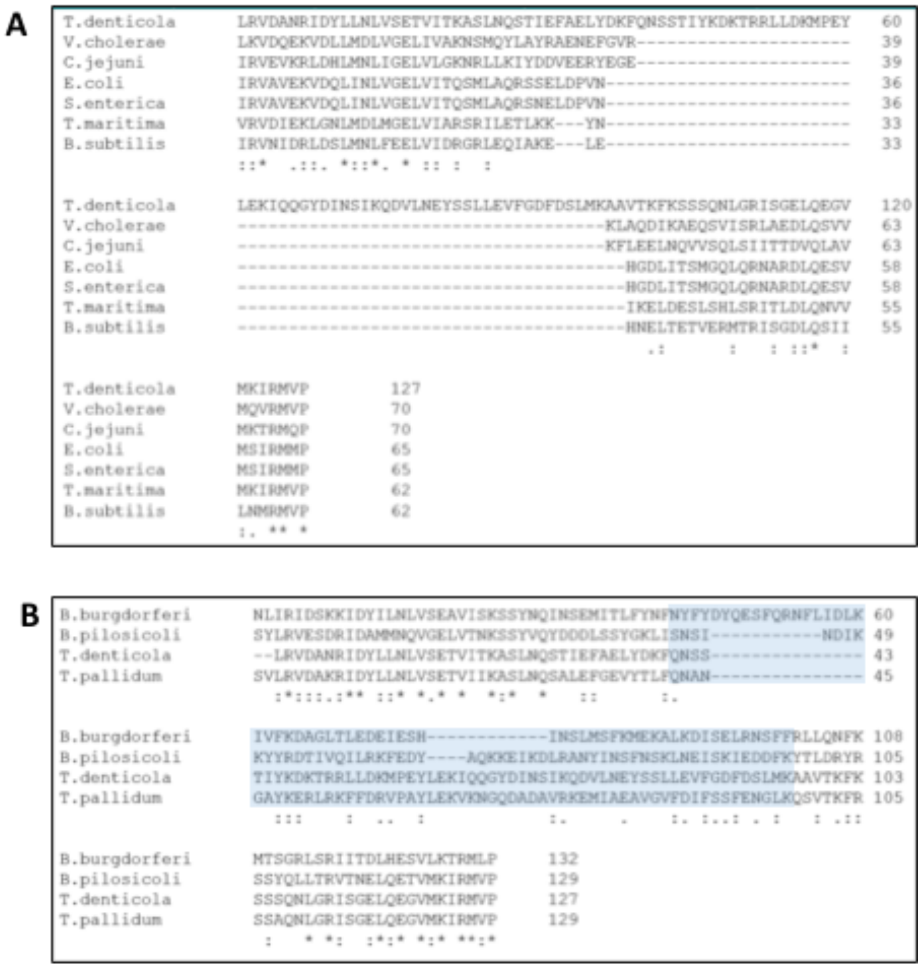

Fig. S10 Multiple sequence alignments of the CheA P3 domain from several bacteria demonstrates the presence of additional P3 residues in *Td* and other Spirochetes. (A) CheA P3 alignments of *Td* and P3 from other bacteria with previously characterized chemotaxis proteins. *Td* possesses ~50 residues that are not found in the other homologs and are located in between the traditional dimerization helices. (B) *Td* CheA P3 aligned with other Spirochete P3. The additional residues identified in alignment **A** are highlighted in blue. Figures were made using Clustal Omega.

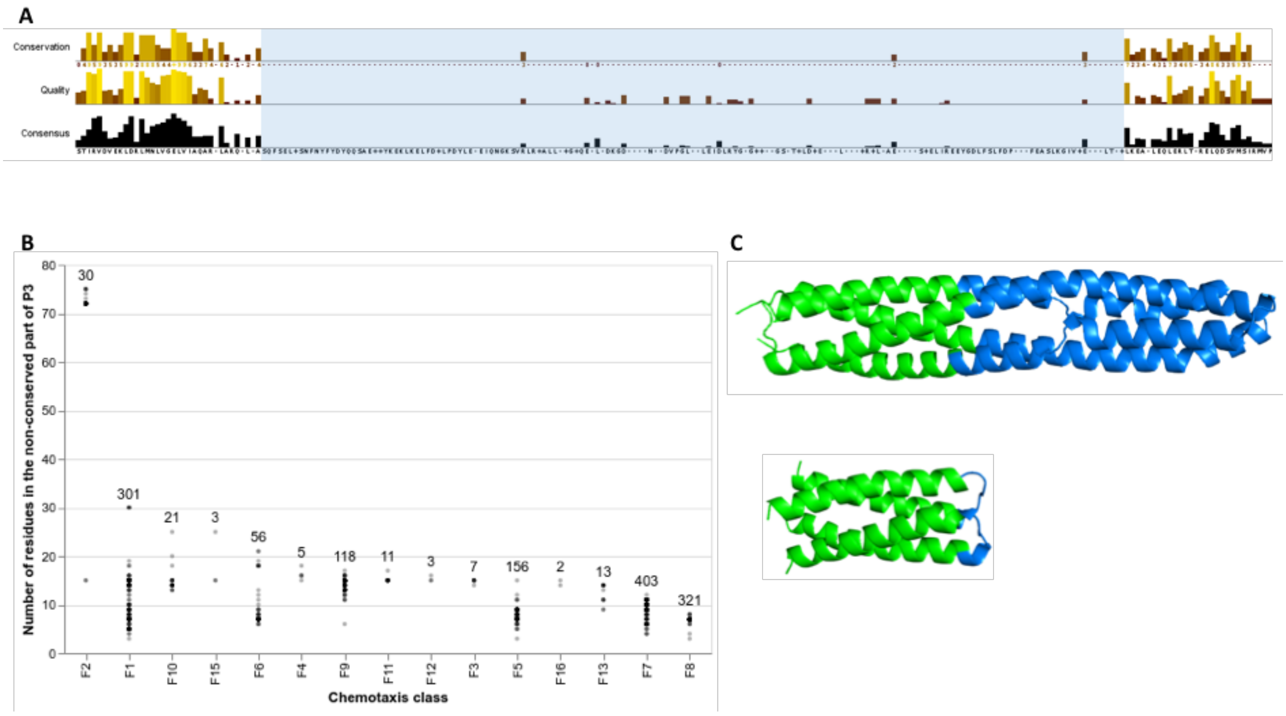

Fig. S11 Analysis of non-redundant CheA P3 domains with a 75% sequence identity cut-off (1450 sequences). (A) Conservation scores of the P3 alignment with the 1450 sequences. The traditional P3 helices are largely conserved, but regions located between the helices are non-conserved. (B) Analyses of CheA from different chemotaxis classes reveals that CheA F2 homologs possess the most residues in the non-conserved region of P3. (C) The non-conserved regions are llustrated with the two known CheA P3 structures. Top: *Td* P3 possesses 71 residues that align to the non-conserved region (PDB ID: 6Y1Y). Bottom: *Tm* P3 possesses seven residues that align to the non-conserved region (PDB ID: 1B3Q).

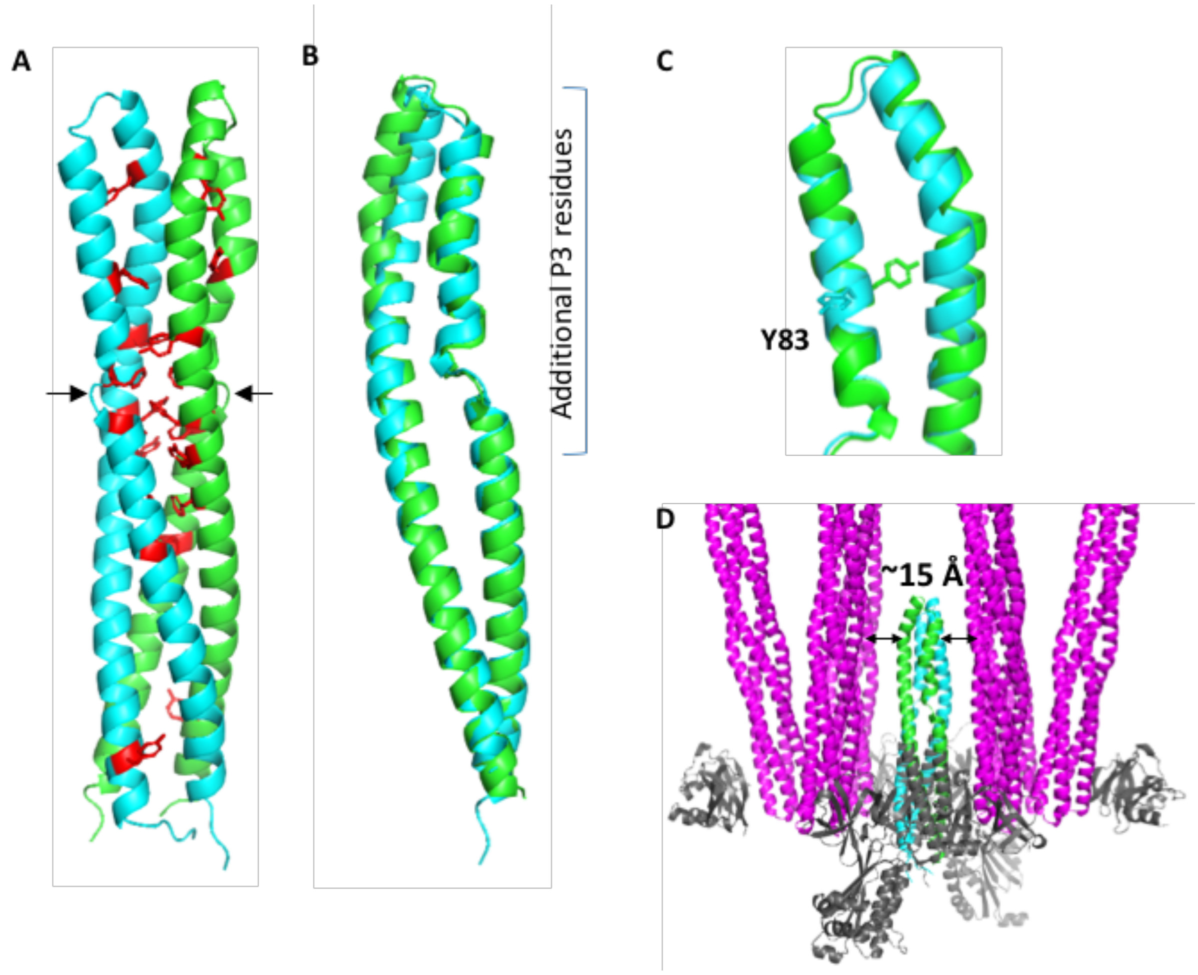

Fig. S12 The P3 domain of *Td* CheA. (A) The CheA P3 dimer contains a cluster of Phe and Tyr residues near the breakages in the helices (black arrows). All Phe and Tyr residues are highlighted in red. (B) The crystal structure of CheA P3 demonstrates asymmetry in the subunits. (C) Repositioning of Y82 in the subunits induces alterations of adjacent residues and may account for subunit asymmetry. (D) Alignment of the P3 structure to a previously determined model of the chemotaxis array in *E. coli* (PDB ID: 3JA6) indicates that the P3 domain lies within ~15 Å of the receptors (when measuring from peptide back-bone). CheA and CheW in this model are shown in grey.

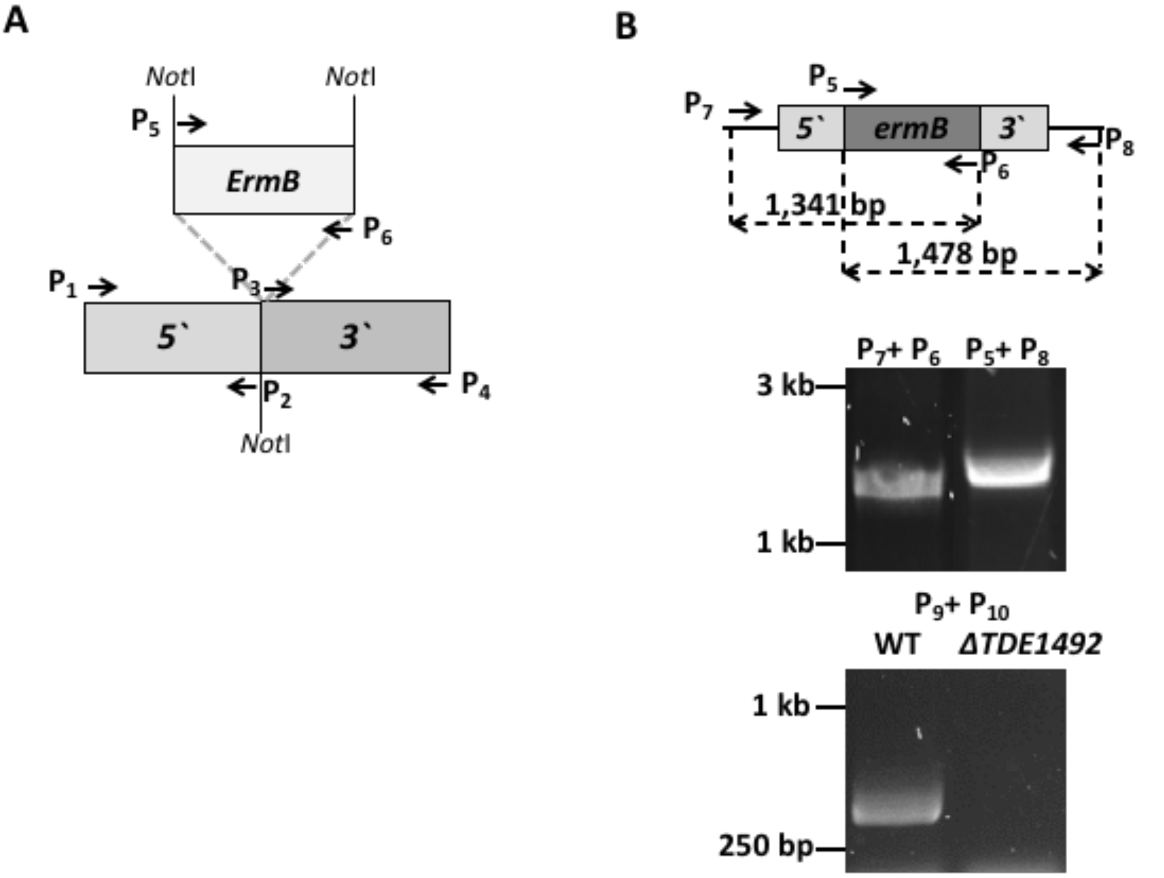

Fig. S13 Diagrams illustrating construction of the *TDE1492::ermB* vector (A) for the targeted mutagenesis of *TDE1492 (781-1,308 nt)* by in-frame replacement of *TDE1492* using *ermB* cassette**.** These constructs were constructed by two-step PCR followed by DNA cloning. Arrows represent the relative positions and orientations of these primers, which are listed in Table S3. *ermB* = erythromycin resistance. (B) Characterization of the *ΔTDE1492* strain by PCR analysis. The top panel illustrates how the PCR analysis is designed; the bottom panel is the PCR results. Arrows represent the relative positions and orientations of these primers; the numbers are predicted sizes of PCR products generated by the corresponding primers. The primer P_7 is located at the 5'-end of *TDE1492*, P_6 at the 3'-end of *ermB*, P_5 at the 5'-end of *ermB*, P_8 at the flanking region of *TDE1492*, P_9 at the middle of *TDE1492*, and P_{10} at the 3'-end of *TDE1492*. The sequences of these primers are listed in Table S3.

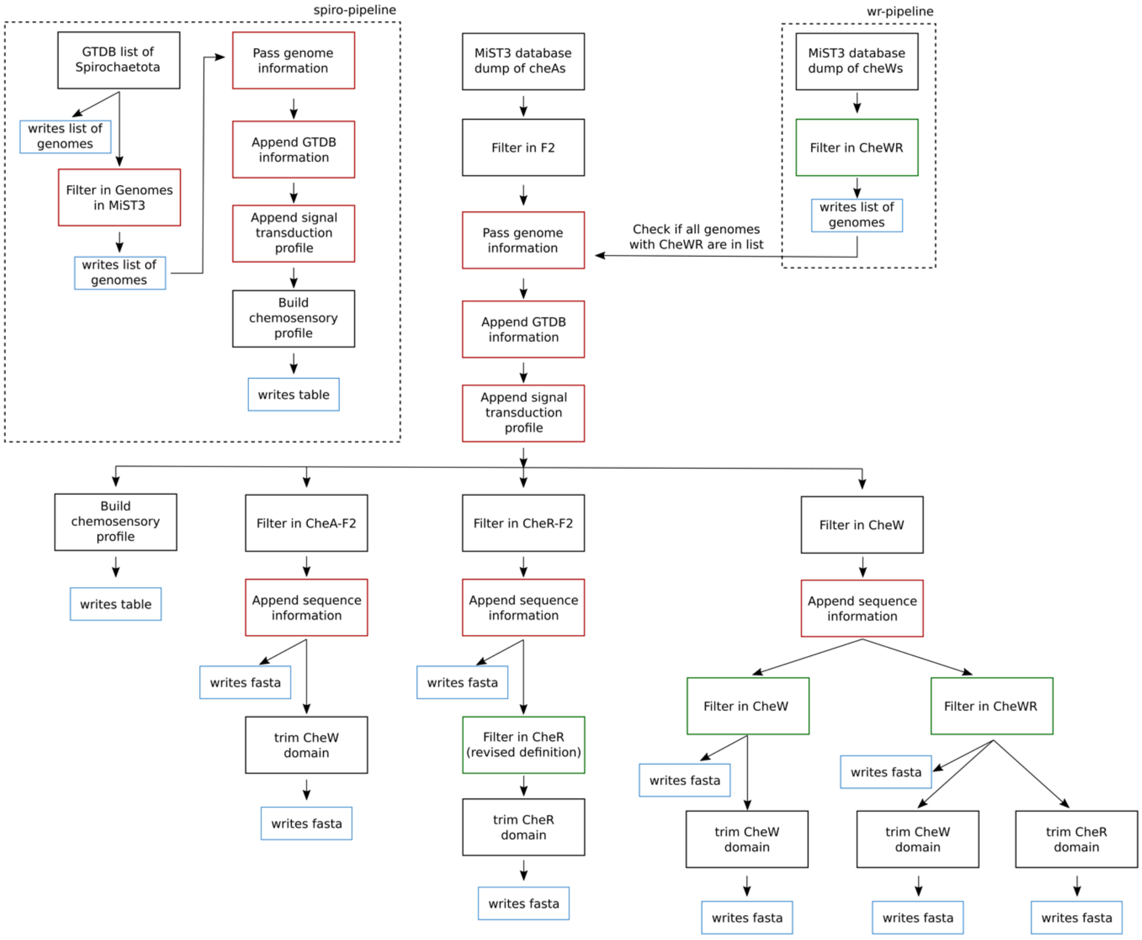

Fig. S14 Flowchart of the three major pipelines used to produce the bioinformatics datasets. Steps marked in red represents fetching information from MiST3 database, in green are steps requiring RegArch as a filter, and in blue indicate writing data to file.

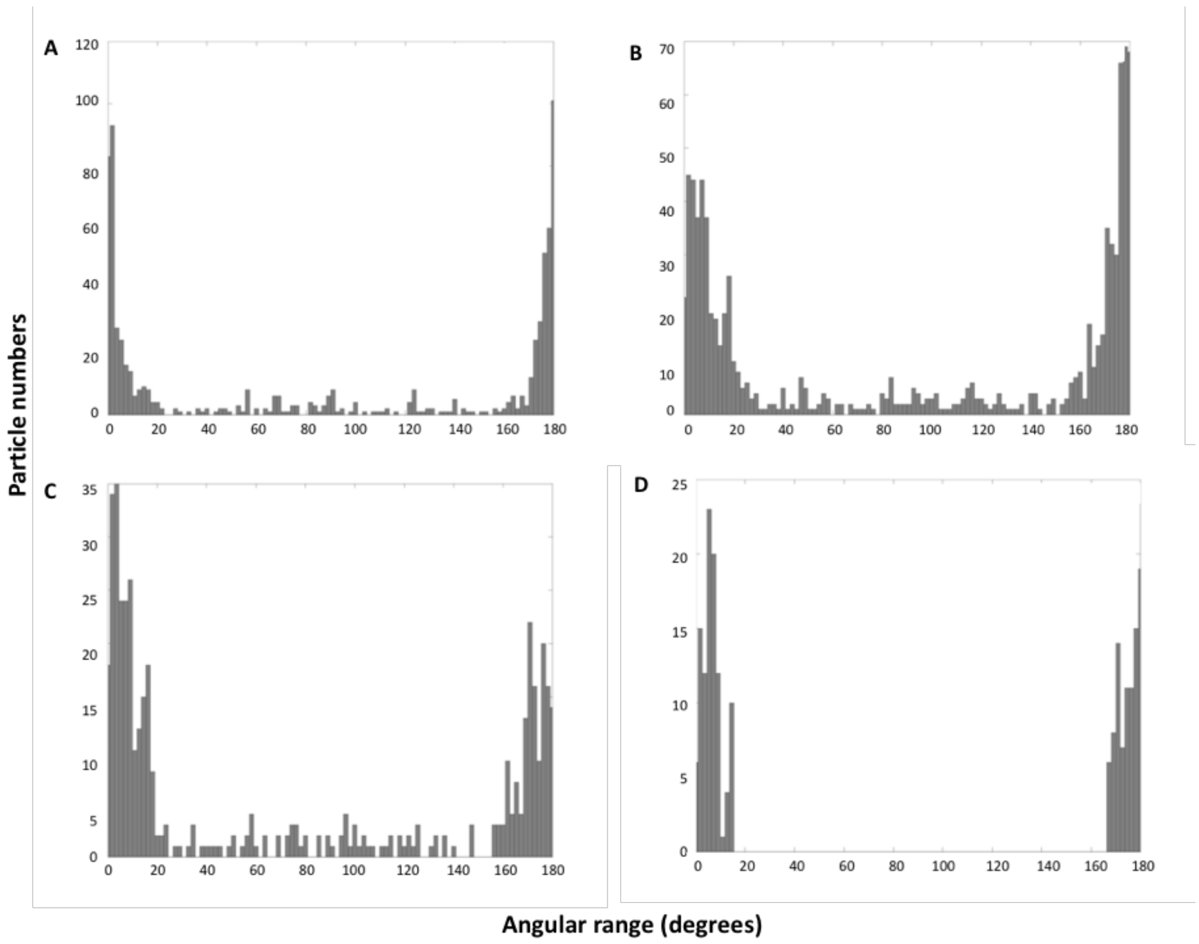

Fig. S15 Angular distribution graphs of the sub-tomogram averages for the WT strain (A), the Δ2498 strain (B), the Δ2498 Δ2496 strain (C), and ΔCheR-like strain (D).

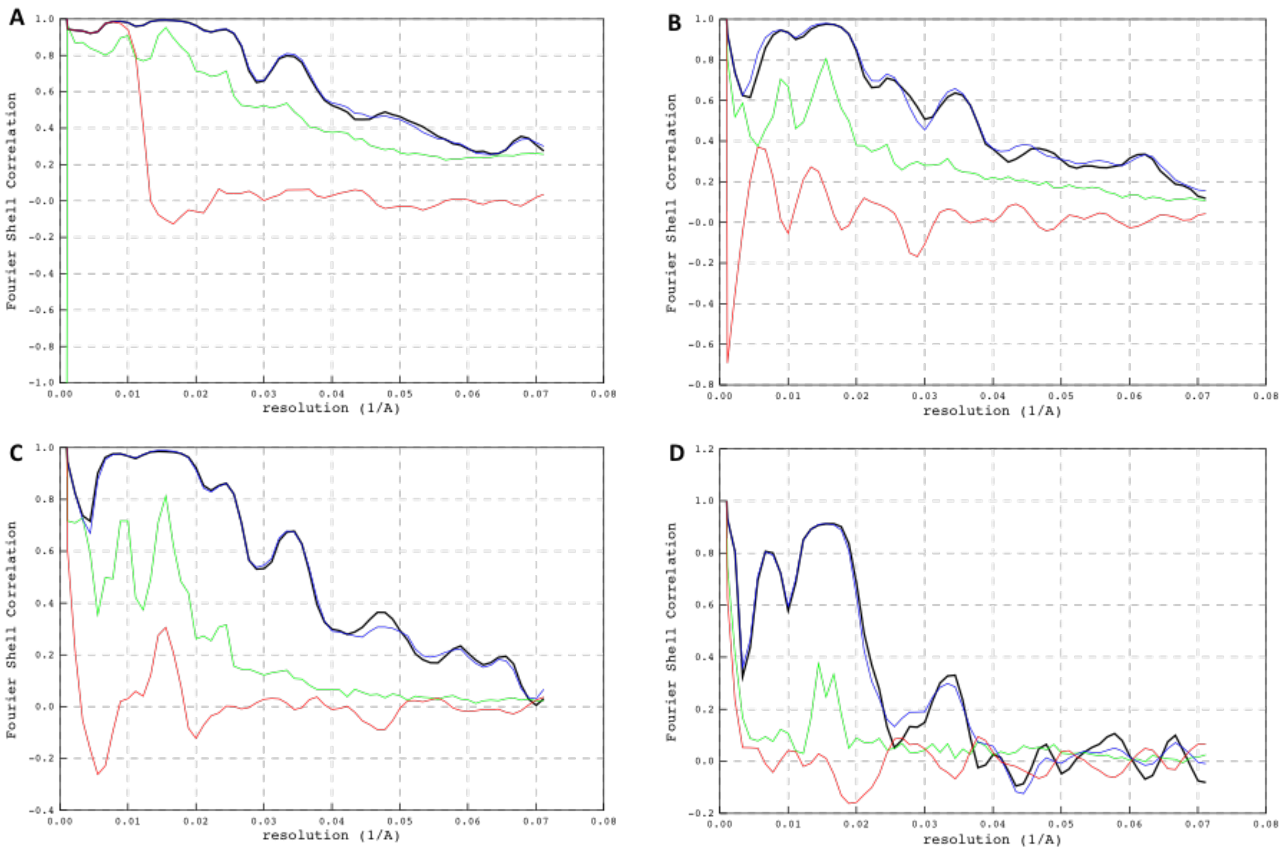

Fig. S16 Fourier shell correlation (FSC) graphs of the sub-tomogram averages for the WT strain (A), the Δ2498 strain (B), the Δ2498 Δ2496 strain (C), and ΔCheR-like strain (D). Blue: FSC of masked maps; Green: FSC of unmasked maps; Black: Corrected FSC; Red: Phase randomized FSC.

Td strain	Average angle (°)
WT (n=9)	9.67 +/- 9.21
Δ2498 (n=9)	9.55 +/- 8.75
Δ2498Δ2496 (n=8)	12.18 +/- 7.62
Combined average:	10.40 +/- 8.59

Table S1A. Statistics for the average angle between the *Td* cell axis and the 'strands' of CheA:CheW rings.

Vc cell number	Inner membrane curvature (/μm)
1	6.2
2	15.4
3	14.2
4	7.4
5	4.3
6	7.5
Average:	9.155 +/- 4.53

Table S1B. Statistics of inner membrane curvature for 6*Vc* mini-cells.

Td cell number	Inner membrane curvature (/μm)	Baseplate curvature (/μm)
1	35.0	49.7
2	25.4	54.0
3	34.2	63.7
4	43.1	96.1
5	30.8	99.7
6	41.1	55.7
7	39.6	74.6
8	33.1	47.7
9	46.2	47.8
10	29.1	67.1
Average:	35.8 +/- 6.6	65.6 +/- 19

Table S1C. Statistics of inner membrane and baseplate curvature for 10 WT *Td* cells.

Primers	Sequences (5'-3')	Note [a]
P_1	CGGGCGTAGGCATCGGAGATAC	5` portion for TDE1492 inactivation; [F]
P_2	TTACAATATCACCTGAGCTGGCGGCCGCTTAAGGTGACA AGAAAGATGATA	5` portion for TDE1492 inactivation; [R]
P_3	TATCATCTTTCTTGTCACCTTAAGCGGCCGCCAGCTCAG GTGATATTGTAA	3` portion for TDE1492 inactivation; [F]
P_4	CCCAGAGCACTTATCATAAC	3` portion for TDE1492 inactivation; [R]
P_5	ATGAACAAAAATATAAAATATTCTC	Erythromycin B cassette (ermB); [F]
P_6	TTATTTCCTCCCGTTAAATAATAG	Erythromycin B cassette (ermB); [R]
P_7	ATGGAAGAAATGAAAGAAC	5` flanking region of TDE1492, ΔTDE1492 PCR analysis; [F]
P_8	GATATAGTTCTTGGCTCCAAG	3` flanking region of TDE1492, ΔTDE1492 PCR analysis; [R]
P_9	GAGGCCTATATAAATGCC	TDE1492, ΔTDE1492 PCR analysis; [F]
P_{10}	CTTGGGCATTGCCTCATTATG	TDE1492, ΔTDE1492 PCR analysis; [R]

Table S3. Oligonucleotide primers used in this study

[a] Underlined sequences are engineered restriction cut sites for DNA cloning; [F] forward; [R] reverse.
