# Supplementary material for: Atypical chemoreceptor arrays accommodate high membrane curvature
Source: Nat Commun. 2020 Nov 13;11:5763. doi: 10.1038/s41467-020-19628-6 (PMC7666581; doi:10.1038/s41467-020-19628-6)
Supplement: Supplementary file 5 — Reporting Summary [file 41467_2020_19628_MOESM5_ESM.pdf]

nature research

Corresponding author(s): Ariane Briegel

Last updated by author(s): Oct 22, 2020

Reporting Summary

Nature Research wishes to improve the reproducibility of the work that we publish. This form provides structure for consistency and transparency in reporting. For further information on Nature Research policies, see Authors & Referees and the Editorial Policy Checklist.

Statistics

For all statistical analyses, confirm that the following items are present in the figure legend, table legend, main text, or Methods section.

n/a	Confirmed	
☐	☒	The exact sample size (n) for each experimental group/condition, given as a discrete number and unit of measurement
☐	☒	A statement on whether measurements were taken from distinct samples or whether the same sample was measured repeatedly
☐	☒	The statistical test(s) used AND whether they are one- or two-sided<br>*Only common tests should be described solely by name; describe more complex techniques in the Methods section.*
☒	☐	A description of all covariates tested
☒	☐	A description of any assumptions or corrections, such as tests of normality and adjustment for multiple comparisons
☒	☐	A full description of the statistical parameters including central tendency (e.g. means) or other basic estimates (e.g. regression coefficient) AND variation (e.g. standard deviation) or associated estimates of uncertainty (e.g. confidence intervals)
☒	☐	For null hypothesis testing, the test statistic (e.g. F, t, r) with confidence intervals, effect sizes, degrees of freedom and P value noted<br>*Give P values as exact values whenever suitable.*
☒	☐	For Bayesian analysis, information on the choice of priors and Markov chain Monte Carlo settings
☒	☐	For hierarchical and complex designs, identification of the appropriate level for tests and full reporting of outcomes
☒	☐	Estimates of effect sizes (e.g. Cohen's d, Pearson's r), indicating how they were calculated

Our web collection on statistics for biologists contains articles on many of the points above.

Software and code

Policy information about availability of computer code

Data collection	For the bioinformatics analyses, we built custom scripts using TypeScript-3.7.5 and NodeJS-12.13. These scripts are available to editors and reviewers upon request, and will be publicly available on GitHub upon publication. We also used packages publicly available at the node package manager repository (NPM): RegArch-1.0.1, gtdb-local-0.0.12, Phylogician-TS-0.10.1-4, BioSeq-TS-0.2.4, MiST3-TS-0.7.6.<br><br>SerialEM 3.7 was used to collect the cryo-ET data.
Data analysis	For the bioinformatics analyses we used the following: L-INS-I algorithm from the MAFFT package, CD-HIT v4.6, Jalview, RAxML v8.2.10, TreeCollapseCL4, Weblogo 3.7.<br>Dynamo 1.380 was used for cryo-ET particle selection and averaging.

For manuscripts utilizing custom algorithms or software that are central to the research but not yet described in published literature, software must be made available to editors/reviewers. We strongly encourage code deposition in a community repository (e.g. GitHub). See the Nature Research guidelines for submitting code & software for further information.

Data

Policy information about availability of data

All manuscripts must include a data availability statement. This statement should provide the following information, where applicable:

- Accession codes, unique identifiers, or web links for publicly available datasets
- A list of figures that have associated raw data
- A description of any restrictions on data availability

The cryo-ET sub-tomogram averages that support these findings are deposited in the Electron Microscopy Data Bank (EMDB) with accession codes EMD-10784, EMD-10785, EMD-10786, EMD-10787, EMD-10788, EMD-10790. Images of these cryo-ET averages correspond to Figures 1, 2, S5, S7, S10. The protein x-ray crystallography structure that supports these findings is deposited in the Protein Data Bank (PDB) with accession code 6Y1Y. The figures corresponding to the x-ray

crystallography data are Figures 4, S13, S14. All depositions will be publicly available with publication.

Field-specific reporting

Please select the one below that is the best fit for your research. If you are not sure, read the appropriate sections before making your selection.

☒ Life sciences ☐ Behavioural & social sciences ☐ Ecological, evolutionary & environmental sciences

For a reference copy of the document with all sections, see nature.com/documents/nr-reporting-summary-flat.pdf

Life sciences study design

All studies must disclose on these points even when the disclosure is negative.

Sample size	For each Treponema denticola strain, cryo-ET data for ~30 cells were selected for collection. Selection was based on the quality of cell freezing/clarity. Of those cells, the cells that possess visible chemotaxis array top views were used for sub-tomogram averaging. All sub-tomograms were used to generate the final averages.
Data exclusions	No data were excluded from the analysis.
Replication	For all Treponema denticola strains, 30 cells were selected for cryo-ET and the generated sub-tomogram averages reflect particle averages (194-894 particles per strain) for all cells that possess top view arrays (5-10 cells per strain).
Randomization	N/A
Blinding	N/A

Reporting for specific materials, systems and methods

We require information from authors about some types of materials, experimental systems and methods used in many studies. Here, indicate whether each material, system or method listed is relevant to your study. If you are not sure if a list item applies to your research, read the appropriate section before selecting a response.

Materials & experimental systems

n/a	Involved in the study	
☒	☐	Antibodies
☒	☐	Eukaryotic cell lines
☒	☐	Palaeontology
☒	☐	Animals and other organisms
☒	☐	Human research participants
☒	☐	Clinical data

Methods

n/a	Involved in the study	
☒	☐	ChIP-seq
☒	☐	Flow cytometry
☒	☐	MRI-based neuroimaging
